# Intelligent-Responsive Enrofloxacin-Loaded Chitosan Oligosaccharide–Sodium Alginate Composite Core-Shell Nanogels for On-Demand Release in the Intestine

**DOI:** 10.3390/ani12192701

**Published:** 2022-10-08

**Authors:** Wanhe Luo, Mujie Ju, Jinhuan Liu, Samah Attia Algharib, Ali Sobhy Dawood, Shuyu Xie

**Affiliations:** 1 Engineering Laboratory for Tarim Animal Diseases Diagnosis and Control, College of Animal Science and Technology, Tarim University, Alar 843300, China; 2 Key Laboratory of Tarim Animal Husbandry & Science Technology, Xinjiang Production & Construction Corps, Alar 843300, China; 3 Department of Clinical Pathology, Faculty of Veterinary Medicine, Benha University, Moshtohor, Toukh 13736, Egypt; 4 National Reference Laboratory of Veterinary Drug Residues (HZAU), Huazhong Agricultural University, Wuhan 430070, China; 5 Infectious Diseases, Faculty of Veterinary Medicine, University of Sadat City, Sadat City 32897, Egypt

**Keywords:** enrofloxacin, intestinal infection, nanogels, *Escherichia coli* (*E. coli*), on-demand release

## Abstract

**Simple Summary:**

Novel pharmaceutical formulations are attracting interest in their potential to overcome the poor palatability and strong gastric irritation of enrofloxacin. To overcome the difficulty of treating intestinal *Escherichia coli* infections, an oral intelligent-responsive chitosan-oligosaccharide (COS)–sodium alginate (SA) composite core-shell nanogel loaded with enrofloxacin was designed and systematically evaluated. Scanning electron microscopy images revealed that enrofloxacin nanogels were incorporated into the nano-sized cross-linked networks. The physical state and molecular interaction among the components of the nanogel and the enrofloxacin were evaluated by Fourier transform infrared spectroscopy. Furthermore, their biocompatible structure, high drug loading efficacy, ideal stability, “on-demand” release at the target site, and antibacterial activity were confirmed. Thus, the present study may serve as a fruitful platform to explore nanogel to resolve the challenge of enrofloxacin formulation development and the fight against intestinal bacterial infections.

**Abstract:**

Enrofloxacin has a poor palatability and causes strong gastric irritation; the oral formulation of enrofloxacin is unavailable, which limits the treatment of *Escherichia coli* (*E. coli*) infections via oral administration. To overcome the difficulty in treating intestinal *E. coli* infections, an oral intelligent-responsive chitosan-oligosaccharide (COS)–sodium alginate (SA) composite core-shell nanogel loaded with enrofloxacin was explored. The formulation screening, characteristics, pH-responsive performance in gastric juice and the intestinal tract, antibacterial effects, therapeutic effects, and biosafety level of the enrofloxacin composite nanogels were investigated. The optimized concentrations of COS, SA, CaCl_2_, and enrofloxacin were 8, 8, 0.2, and 5 mg/mL, respectively. The encapsulation efficiency, size, loading capacity, zeta potential, and polydispersity index of the optimized formulation were 72.4 ± 0.8%, 143.5 ± 2.6 nm, 26.6 ± 0.5%, −37.5 ± 1.5 mV, and 0.12 ± 0.07, respectively. Scanning electron microscopy images revealed that enrofloxacin-loaded nanogels were incorporated into the nano-sized cross-linked networks. Fourier transform infrared spectroscopy showed that the nanogels were prepared by the electrostatic interaction of the differently charged groups (positive amino groups (-NH_3_^+^) of COS and the negative phenolic hydroxyl groups (-COO^−^) of SA). In vitro, pH-responsive release performances revealed effective pH-responsive performances, which can help facilitate targeted “on-demand” release at the target site and ensure that the enrofloxacin has an ideal stability in the stomach and a responsive release in the intestinal tract. The antibacterial activity study demonstrated that more effective bactericidal activity against *E. coli* could have a better treatment effect than the enrofloxacin solution. Furthermore, the enrofloxacin composite nanogels had great biocompatibility. Thus, the enrofloxacin composite core-shell nanogels might be an oral intelligent-responsive preparation to overcome the difficulty in treating intestinal bacterial infections.

## 1. Introduction

Intestinal bacterial infections pose a serious threat to animal health, which has aroused considerable concern [1]. In particular, *Escherichia coli* (*E. coli*) infections are of great importance in the global medical and veterinary community [2,3]. Because the site of *E. coli* infections is in the intestine, the best treatment is oral administration. Enrofloxacin is used to treat various animal infections due to its powerful antibacterial properties, e.g., *E. coli* infections. However, enrofloxacin has a poor palatability and causes strong gastric irritation. The oral formulation of enrofloxacin for swine is not available, which limits the treatment of *E. coli* infections in pigs via oral administration [4]. Masking the taste and reducing gastric irritation in intelligent oral delivery systems will improve the therapeutic effect and increase compliance through on-demand release at the site of intestinal infections. Therefore, the design of intelligent oral delivery systems for enrofloxacin is imperative.

Nanogels are mainly characterized by excellent structural stability, excellent palatability, and strong antibacterial activity of loaded antibiotics against target bacteria [5,6]. Taking into account the micro-environment of different gastrointestinal tracts (e.g., different pH values), an on-demand delivery can be permitted by the smart responsive nanogels, which are accessible at different pHs of the gastrointestinal tract parts [7]. Sodium alginate (SA), a hydrogel material and drug carrier, has received a lot of attention due to its unchanged characteristics in gastric juice [8]. This feature can ensure that the drug passes through the stomach completely. What is more, SA nanogels can rapidly swell in the intestinal tract and enlarge the sizes of the nanogels, so that the drug can enable on-demand release in the intestine [9]. Chitosan oligosaccharide (COS) is a nanogel material with a proton sponge effect, which makes it a promising carrier for controlling drug release and achieving sustained release under the condition of a weak acid or weak base [10,11].

**Figure 1:**
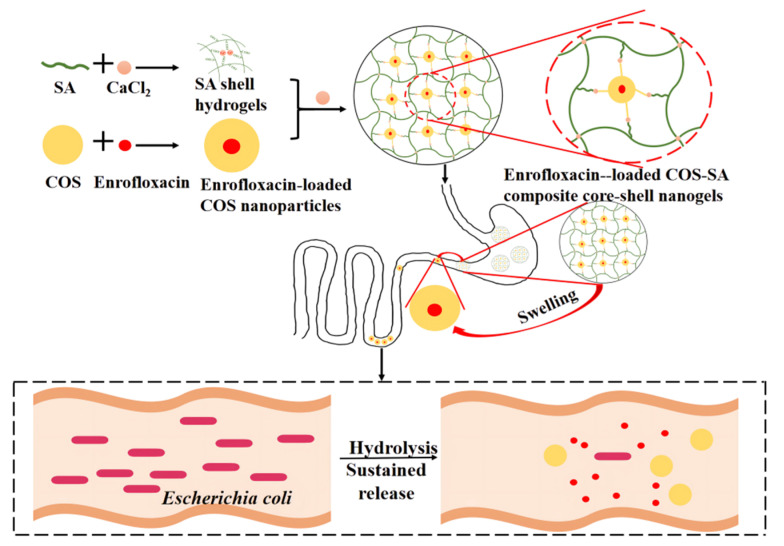
Diagrammatic sketch of enrofloxacin-loaded chitosan oligosaccharide–sodium alginate composite core-shell nanogels with the on-demand release of enrofloxacin for intestinal *E. coli* infections.

On this basis, COS can ensure the sustained release of enrofloxacin in the intestinal tract. In view of this, enrofloxacin may be incorporated into the crosslinked network of COS to form enrofloxacin-loaded COS nanoparticles. SA crosslinking with Ca^2+^ could form uniformly excellent SA shell hydrogels. Subsequently, the enrofloxacin-loaded COS nanoparticles can be encapsulated in SA hydrogel shells, forming COS–SA composite core-shell nanogels loaded with enrofloxacin. SA shell hydrogels can ensure the integrity of the enrofloxacin and are not destroyed by gastric acid. When the enrofloxacin composite nanogels reach the intestine, the SA hydrogel will rapidly swell in the intestinal tract and the enrofloxacin-loaded COS nanoparticles will be released, thus effectively treating *E. coli* infections by the on-demand release effect (Figure 1). The formulation screening, characteristics, palatability, pH-responsive performance, in vitro release in gastric juice and the intestinal tract, antibacterial action, treatment efficacy, and biosafety of enrofloxacin composite core-shell nanogels were explored. The enrofloxacin composite core-shell nanogels will improve the therapeutic treatment of *E. coli* infections through their on-demand release in the intestine and excellent palatability.

## 2. Material and Methods

### 2.1. Material

Enrofloxacin solution, enrofloxacin, and enrofloxacin standard were obtained by Dingyuan Co., Ltd. (Alar, China), Wuhan Animal Pharmaceutical Co., Ltd. (Wuhan, China), and the China Institute of Veterinary Drugs Control (Beijing, China), respectively. COS, SA, and calcium chloride (CaCl_2_, ≥93.0%) were purchased from Dingyuan Co., Ltd. (China). The *E. coli* ATCC 25922 was provided by the Engineering Laboratory for Tarim Animal Diseases Diagnosis and Control of Tarim University (Alar, China).

### 2.2. Preparation

The COS–SA composite core-shell nanogels loaded with enrofloxacin were prepared according to previous reports [12,13,14]. Briefly, SA (100, 200, or 400 mg) and 25 mL ultrapure water were added together to form a suspension under a magnetic stirrer (HSC-19T, Qun’an Experimental Instrument Co., Ltd., Huzhou, China). Then, 1 mL CaCl_2_ (5, 10, or 20 mg/mL) was added drop by drop to the SA solution to form SA hydrogels. Simultaneously, COS (200, 400, or 600 mg) was dissolved in 15 mL of high-purity water. Then, 9 mL of 0.1 mol/L NaOH containing enrofloxacin (250, 500, or 750 mg) was added to the COS mixture, forming the COS nanoparticles loaded with enrofloxacin. Finally, the enrofloxacin-loaded COS nanoparticles were added dropwise to the SA hydrogels forming the COS–SA composite core-shell nanogels loaded with enrofloxacin. The optimal concentrations of SA and COS were determined by the encapsulation efficiency (EE) and loading capacity (LC). Briefly, the enrofloxacin composite nanogels with different formulations were centrifugated at 14,000 RPM for 60 min, and then the enrofloxacin in the supernatant was measured to calculate EE. The precipitate was lyophilized into powder after re-suspending to calculate LC. Each sample preparation was repeated three times. The EE and LC were defined as follows [14]:

EE (%) = [(Weight of enrofloxacin added−weight of enrofloxacin in supernatant)/(Weight of enrofloxacin added)] × 100%

LC (%) = [(Weight of enrofloxacin added−weight of enrofloxacin in supernatant)/(Weight of enrofloxacin composite nanogels)] × 100%

### 2.3. Characterization

#### 2.3.1. Surface Morphology

After preparing optimal enrofloxacin nanogels, the appearance images were observed. Subsequently, they were freeze-dried using the FDU-1200 lyophilizer (Tokyo Physicochemical Instruments Co., Ltd, Tokyo, Japan) at the condensation temperature (−80 °C) for 8–10 h and photographed by optical microscopy (LJ-CLP03, Shenzhen Longji Instrument Equipment Co., Ltd, Shenzhen, China). Briefly, when the lyophilized sample was placed on a glass slide, the LJ-CLP03 optical microscopy was used to observe its morphology. More importantly, their characterization was determined using Hitachi S-4800 scanning electron microscopy (SEM), HITACHI, Tokyo, Japan). Briefly, 1 mg enrofloxacin composite nanogels suspended in 1 mL distilled water and 2 μL of the suspension were placed on a coverslip. The samples were coated with gold using an ion sputter and examined at an accelerating voltage of 20 kV after oven-drying.

#### 2.3.2. Zeta Potential (ZP), Polydispersity Index (PDI), and Particle Diameter

After the enrofloxacin nanogels were diluted 100 times, the Zeta ZX3600 (Malvern Instruments, Malvern, UK) was used to determine the mean particle size, ZP, and PDI [14].

#### 2.3.3. Fourier Transform Infrared (FTIR)

After the enrofloxacin composite nanogels were freeze-dried, they and their different components were determined by FTIR spectrophotometer (Nicolet iS50, Thermo Scientific Inc., Waltham, MA, USA). Briefly, the dried nanogels, after being converting into a fine powder, were added to form a disc. Afterward, each sample was estimated by Carson apodization 32 times at a resolution of 4 cm^−1^.

### 2.4. pH-Responsive Performances

The simulated gastric juice and intestinal fluid were prepared as described previously [4]. Briefly, 7 mL of concentrated hydrochloric acid was dissolved in enough water, and 2.0 g NaCl was added to 3.2 g pepsin with complete dissolution to obtain simulated gastric juice (pH 1.2). Simultaneously, 6.8 g potassium dihydrogen phosphate, 10.0 g trypsin, and 77 mL sodium hydroxide solution (0.2 molar/L) were added in water to simulated intestinal juice (pH 7.4). The pH was measured using a pH meter. In addition, the pH of the original enrofloxacin composite nanogels was also measured. To evaluate the pH-responsive performance of the enrofloxacin composite nanogels, the formula was exposed to the simulated gastric juice and intestinal fluid. After that, the particles’ diameters, ZP, and PDI were measured. Additionally, the morphological changes in the enrofloxacin composite nanogels in response to the simulated gastric juice and intestinal fluid were observed by SEM. On the other hand, the enrofloxacin-loaded nanogels were added to a dialysis bag (MW: 3500) and then were placed into 500 mL simulated gastric juice (pH 1.2) and simulated intestinal fluid (pH 7.4) at 37 ± 0.5 °C after being sealed in a dissolution tester (RC806, Guangzhou Hangxin Scientific Instrument Co., Ltd, Guangzhou, China). Eventually, 1 mL of the dialysate was used to determine the concentration of enrofloxacin at 10, 20, 30, 60, and 90 min. When 1 mL of dialysate was removed, it was replaced by 1 mL of SGF/SIF to keep the concentration/volume constant in the dialysis bag. Meanwhile, the cumulative curve was calculated according to the cumulative release percentage.

### 2.5. Antibacterial Activity Studies

#### 2.5.1. Broth Microdilution Method

The antibacterial activity of enrofloxacin-loaded COS–SA composite nanogels was measured by the broth microdilution procedure. Briefly, the serial dilution of enrofloxacin composite nanogels using Mueller–Hinton (MH) broth was conducted. In order to ensure the stability of the enrofloxacin composite nanogels, the pHs (7.2) of MH broth and the nanogels were kept equal. Subsequently, the *E. coli* ATCC 25922 was inoculated into each tube to achieve a final inoculum of 1 × 10^6^ CFU/mL. The minimum inhibitory concentration (MIC) is the lowest concentration required for inhibiting the visible bacterial growth after 24 h incubation of the bacterial cultures at 37 °C [15,16,17].

#### 2.5.2. Time-Kill Curves

In this study, the in vitro kill curves of enrofloxacin nanogels using *E. coli* ATCC 25922 were calculated by considering time as a function of log_10_ CFU/mL. Briefly, after 1 mL MH cultured broth with *E. coli* (10^6^ CFU/mL) was obtained, enrofloxacin nanogels were used to create a serial dilution (1/2 × MIC, 1 × MIC, 2 × MIC, and 4 × MIC). The tubes were incubated at 37 °C, and the bacterial count (CFU/mL) was calculated with the help of the agar dilution method with incubating tubes for 1, 2, 4, 8, 12, 24, 48, and 72 h. Briefly, 100 µL of bacteria were coated on MH agar. After 18–24 h, the number of bacteria on MH agar was calculated [18].

### 2.6. Animal Experiment

#### 2.6.1. Mouse Infection Model

The mouse bacterial infection model was constructed using *E. coli* ATCC 25922 [19]. Concisely, 12 healthy Kunming mice (18–23 g) were randomly grouped into two sets (six mice per group). Each animal was given 0.1 mL of *E. coli* 25,922 suspensions (1.2 × 10^9^ CFU/mL) orally by gavage to structure the infection model. On the other hand, each mouse of the second group was fed with 0.1 mL of 0.9% sodium chloride solution as a control group model. After 24 h, all mice were euthanized, and the intestinal tract was aseptically gathered. The successful settlement of the mouse infection model for each group was assessed using hematoxylin and eosin (H&E) staining [20,21]. In this study, the use of mice and all the experimental protocols concerning the handling of mice followed the requirements of the Experimental Animal Ethics/Ethics Committee at Animal Science Academy of Huazhong Agricultural University and were approved by the Institutional Animal Care and Use Committee at Tarim University (approved number: HZAUSW-2019-009).

#### 2.6.2. Treatment Schedules

Twenty-four Kunming mice (18–22 g) were randomly separated to form four sets (six animals per group): NC group (non-infected control group, which was treated with 0.9% NaCl solution); IC group (infected control group, which was treated with *E. coli*); Native 1 group (infected *E. coli* group, which was treated with enrofloxacin solution); and Native 2 group (infected *E. coli* group, which was treated with enrofloxacin nanogels). Treatment of the groups began 3 d post-*E. coli* infection, then all animals were euthanized on the 7th d after *E. coli* infection [19]. Subsequently, the intestinal tracts of the mice were collected. The therapeutic effects of enrofloxacin nanogels and enrofloxacin solution against *E. coli* infections were compared by H&E staining.

### 2.7. Safety Evaluation

On the 7th day, the renal and liver functions of mice in the NC group, the Native 1 group, and the Native 2 group were evaluated by H&E staining as well as blood biochemical analysis. Briefly, after the intestines of mice were taken, fixed, washed, dehydrated, embedded, and sectioned, an optical microscope was used to observe and take photographs. Simultaneously, 1 mL blood of mice was taken for blood biochemistry analysis, such as alanine aminotransferase (ALT), aspartate aminotransferase (AST), creatinine (CREA), and blood urea nitrogen (BUN).

### 2.8. Statistical Analysis

The experimental data are presented as the mean ± SD and analyzed by one-way analysis of variance (ANOVA) using SPSS software (version 19.0, IBM, New York, USA); results were considered significant at *p* values < 0.05.

## 3. Results and Discussion

### 3.1. Optimization of Formula

In the current study, the enrofloxacin-loaded COS–SA compound core-shell nanogel was optimized using a single factor test with the help of EE and LC as the assessment indicators. It can be found that different concentrations of COS, SA, CaCl_2_, and enrofloxacin all affected the mean EE and LC of the COS–SA composite core-shell nanogels loaded with enrofloxacin (Table 1). The mean LC and EE of the most favorable enrofloxacin composite nanogels were 26.6 ± 0.5% and 72.4 ± 0.8%, respectively. Hence, the optimized concentrations of COS, SA, CaCl_2_, and enrofloxacin were 8, 8, 0.2, and 5 mg/mL, respectively.

### 3.2. Properties

**Figure 2:**
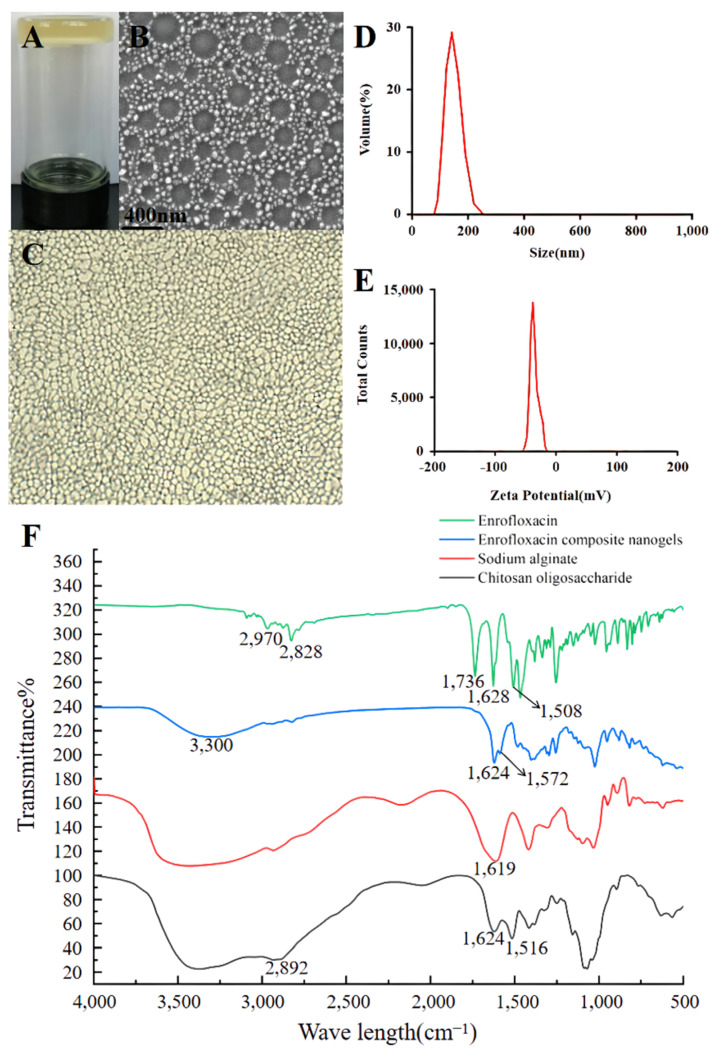
Properties of the optimal enrofloxacin nanogels’ preparation. (**A**) Appearance, (**B**) images of scanning electron microscopy, (**C**) lyophilized nanogels (100× Magnification), (**D**) size range distribution, (**E**) Zeta potential, and (**F**) Fourier transform infrared.

The COS–SA composite core-shell nanogels loaded with enrofloxacin were a homogenous, dark yellow suspension in an upside-down bottle (Figure 2A). When the enrofloxacin-loaded COS was incorporated into the SA solution, cross-linked hybrid nanogels were formed and the enhanced viscoelastic properties were observed. More interestingly, the lyophilized enrofloxacin nanogels still showed uniform cross-linking polymeric networks (Figure 2B). It is noteworthy that the particle size of drugs is one of the important factors affecting the entry of the drugs into bacteria. The smaller the particle size and the larger the surface area of the drugs, the stronger their antibacterial activity. Generally, a particle size of 10–500 nm is ideal [22,23]. Particle charge is one of the factors affecting the stability of drugs. When the ZP of the drug is >+30 mV or <−30 mV, its physical stability is ideal [23]. PDI is often used to evaluate the homogeneity of drugs. When the PDI is closer to zero, it becomes more homogenous [23]. In this study, the mean PDI, sizes, and ZP of the optimum enrofloxacin composite nanogels were 0.12 ± 0.07, 143.5 ± 2.6 nm (Figure 2D), and −37.5 ± 1.5 mV (Figure 2E), respectively. Hence, the enrofloxacin composite nanogels had ideal size fastness and homogeneity. The SEM revealed that the enrofloxacin composite nanogels were double-layer structures and the core SA nanoparticles were covered by a nano-sized crosslinked COS network (Figure 2C). This phenomenon is related to the robust ionic cross-linking of the SA-forming molecules and the CaCl_2_ molecules, the electric charges interaction amongst the positively charged amino groups (-NH_3_^+^) in the COS molecules, and the negatively charged phenolic hydroxyl groups (-COO^−^) derived from the SA molecules on the surface of SA nanogels. The previous reactions took place using an ionic crosslinker, as well as the intramolecular or intermolecular hydrogen bond interactions among enrofloxacin, SA, and COS molecules. This result was consistent with the schematic simulating diagram of enrofloxacin composite nanogels (Figure 1). The diameters of the enrofloxacin core-shell nanogels measured by SEM were lower than those tested by the Zeta sizer apparatus. The different values might be related to the difference in the nanomaterials’ physical statuses; the measured diameters using a Zeta sizer were in an aqueous status. As the free water and some hydrated water were exposed to evaporation, the enrofloxacin composite nanogels shrunk, resulting in a decrease in the particle sizes tested by SEM [14].

The FTIR spectra of the COS–SA composite core-shell nanogels loaded with enrofloxacin and their ingredients are displayed in Figure 2F. The characteristic FTIR peaks of enrofloxacin were consistent with those measured in other research [22]. The characteristic peaks at 1619 cm^−1^ (-COO^−^) for SA and the characteristic peaks at 1516 cm^−1^ (-NH_2_) for COS disappeared. Moreover, an amide bond (3300, 1624, and 1572 cm^−1^) was generated in enrofloxacin composite core-shell nanogels. This may be due to the electrostatic interactions between the negatively charged COO^−^ of SA and the positively charged NH_3_^+^ of COS. In addition, through the comparison with SA and the enrofloxacin composite nanogels, the wide peak for SA disappeared. This may be attributed to the strong ionic cross-linking between the SA molecular chains and the CaCl_2_ molecules. More importantly, the characteristic peaks at 2970, 2828, 1736, 1628, and 1508 cm^−1^ for enrofloxacin have disappeared, and the wide peaks for SA and COS have disappeared, indicating the intramolecular or intermolecular hydrogen bond interactions among enrofloxacin, SA, and COS molecules. Thus, the COS–SA composite core-shell nanogels loaded with enrofloxacin were prepared successfully.

### 3.3. pH-Responsive Performances

**Figure 3:**
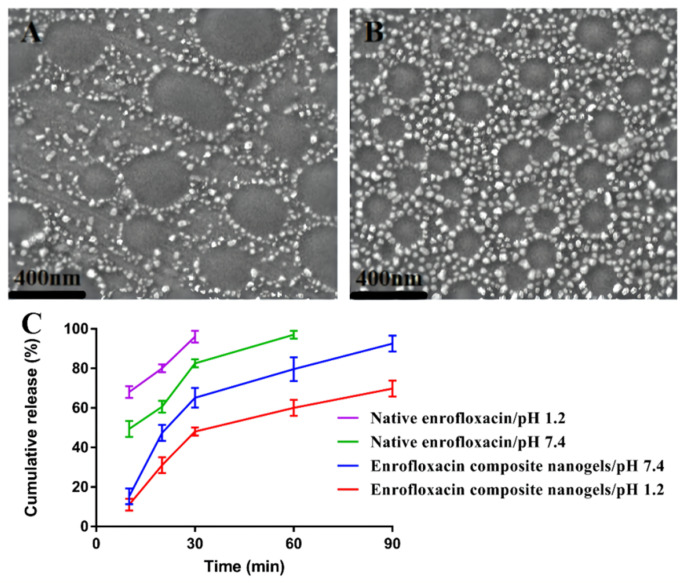
pH-responsive performances of the enrofloxacin-loaded chitosan oligosaccharide–sodium alginate composite core-shell nanogels at distinct pH environments. Scanning electron microscopy of the enrofloxacin nanogels at 7.4 pH (**A**) and 1.2 pH (**B**). (**C**): the accumulative release for the enrofloxacin composite nanogels and native enrofloxacin at pH 1.2 and 7.4.

To evaluate the pH-responsiveness, the responsive changes in the COS–SA composite core-shell nanogels loaded with enrofloxacin in different pHs (1.2 and 7.4 pH values) were studied. With a 7.4 pH, the particle diameters (134.4 ± 3.2 nm) of the enrofloxacin composite nanogels slightly decreased (Table 2). This reduction in nanogels diameters has revealed that the nanoparticles were swelled and dispersed in a neutral medium (Figure 3A). This may be caused by the improvement in the solubility of SA in the case of a neutral pH, resulting in the release of enrofloxacin-loaded COS nanoparticles from the enrofloxacin-loaded COS–SA composite core-shell nanogels [24]. There was no significant change in the size (168.6 ± 4.2 nm) of the enrofloxacin composite core-shell nanogels at a pH 1.2. This may be due to SA enclosing the enrofloxacin-loaded COS nanoparticles and avoiding changing the size of the enrofloxacin-loaded composite nanogels’ particles in gastric juice (Figure 3B). The enrofloxacin composite nanogels had good dispersibility (0.24 ± 0.05) and a high ZP (38.7 ± 0.75 mV) at a pH 1.2. Additionally, the enrofloxacin composite core-shell nanogels at a pH 7.4 had an unsatisfactory dispersibility (0.65 ± 0.03) and an excellent ZP (−36.5 ± 0.43 mV). When the PDI was greater than 0.5, the dispersion had an unsatisfactory dispersibility. The larger the PDI value, the less ideal the dispersion is. Additionally, the enrofloxacin composite nanogels in SEM just showed poor dispersion [23]. The increased PDI value is consistent with the SEM image of a pH 7.4 (Figure 3A). In addition, with the increase in network space structure, a large amount of free water and some hydrated water enters the enrofloxacin composite nanogels, which may lead to a higher swelling rate of the nanogels. More interestingly, the enrofloxacin composite nanogels had no obvious change in pH values (1.2) (Figure 3B). This may be attributed to the function of SA. SA maintains a stable shell hydrogel under acidic conditions (pH 1.2) to ensure that the drug is not damaged by gastric acid. However, under neutral conditions (pH 7.4), SA shell hydrogels are swelled and destroyed, thus releasing the encapsulated drug [25]. The prepared enrofloxacin composite nanogels will not be destroyed in the stomach. However, they swell after entering the intestine, resulting in the release of the encapsulated drug. It also proved the ability of the prepared enrofloxacin-loaded composite nanogels to mask their taste and reduce gastric irritation. Because the enrofloxacin was encapsulated by SA shell hydrogels, the stimulation of gastric acid was effectively avoided [26,27,28].

Considering that the enrofloxacin composite nanogels need to pass through gastric juice and intestinal juice, the release of nanogels in different pHs (1.2 and 7.4) was determined for evaluating the pH-responsive release of the environment (Figure 3C). At a 1.2 pH value, 69.8 ± 2.7% of the enrofloxacin was liberated at 90 min from the COS–SA composite nanogels loaded with enrofloxacin. For native enrofloxacin, 96.4 ± 1.1% of the enrofloxacin was released at 30 min at a pH 1.2. This may be due to the encapsulation of SA delaying the release of enrofloxacin in the enrofloxacin nanogels, and so they have a slightly slower enrofloxacin-loaded COS–SA nanogels release rate compared with that of the enrofloxacin granules prepared by Zhou et al. [4]. At a pH 7.4, 92.5 ± 2.3% was released at 90 min, indicating that COS can ensure the responsive release of enrofloxacin-loaded COS nanoparticles in the intestinal tract. For native enrofloxacin, 81.2 ± 0.4% of the enrofloxacin was released at 30 min in pH 7.4. These findings contribute to the prepared enrofloxacin composite core-shell nanogels treatment of intestinal infections, e.g., *E. coli* infections [29,30,31].

### 3.4. In Vitro Antibacterial Activity Test

**Figure 4:**
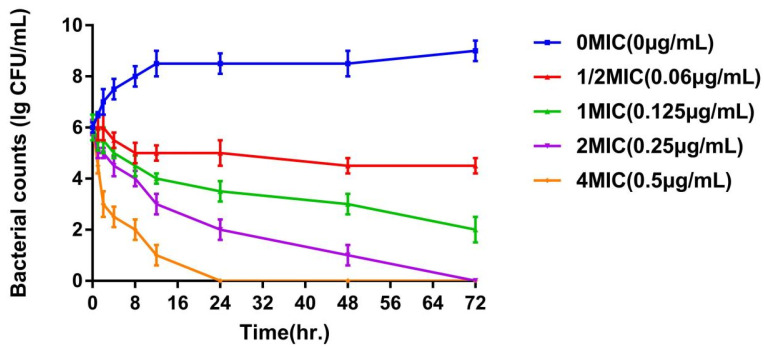
The kill curves of the enrofloxacin composite nanogels against *Escherichia coli* (Mean ± SD, *n* = 3).

The pHs of the MH broth and the enrofloxacin composite nanogels were kept equal (pH = 7.2) to ensure the stability of the enrofloxacin nanogels. The MIC of the enrofloxacin nanogels and enrofloxacin against *E. coli* ATCC 25922 was 0.125 and 1 µg/mL, respectively. The obtained values demonstrated that the enrofloxacin nanogels can improve the antibacterial action of enrofloxacin in the case of *E. coli*. The in vitro time-kill curves of the enrofloxacin composite core-shell nanogels against *E. coli* ATCC 25922 are explained in Figure 4. According to the aforementioned profiles, the enrofloxacin nanogels showed a concentration-dependent gradient bactericidal effect. The more the drug concentrations increase, the more radical and swifter the killing effects. What is more, the bactericidal effect of enrofloxacin was recorded when the enrofloxacin nanogels’ concentration was 2 × MIC (0.25 µg/mL). When the enrofloxacin concentration increased, obvious bacterial growth inhibition was reported in a very short time. Therefore, the bactericidal action increased when increasing the drug concentration. So, the chosen area within the concentration-time curve/MIC (AUC/MIC) could be the most important factor for formulating the dose regimen for the various preparations of enrofloxacin against intestinal bacterial infections caused by *E. coli* due to the concentration-dependent manner of their bactericidal effect [32]. The enrofloxacin nanogels could completely kill the *E. coli* strain. This reported antibacterial potency may be attributed to the effectiveness of the prepared core-shell nanogels for adapting the bioadhesive power of enrofloxacin to the *E. coli* strain, thereby showing a stronger therapeutic efficacy. These conclusions suggested that enrofloxacin composite nanogels might have the potential to figure out the therapeutic obstacles in treating *E. coli* infections.

### 3.5. Therapeutic Effects

**Figure 5:**
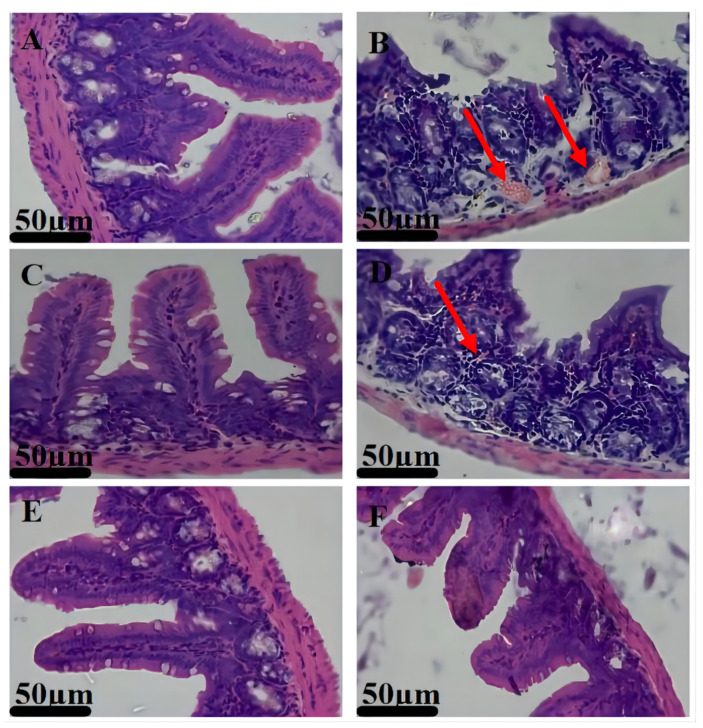
The establishment and therapeutic effect of mouse infection model in different groups. The establishment of the mouse infection model ((**A**): the non-infected control model group; (**B**): the *Escherichia coli* infection model group; the arrow indicates bleeding). The therapeutic effect results were evaluated by H&E staining (**C**): NC group; (**D**): IC group, the arrow indicated inflammatory cell infiltration; (**E**): Native 1 group; (**F**): Native 2 group).

In comparison with the non-infected control model, the outcomes of histopathology proved that the *E. coli* infection model group was established successfully (Figure 5). In comparison with the NC group model, the histopathological results of the *E. coli* infection model group showed that the small intestinal villi were damaged; the arrangement of the epithelial cells was disordered; there was a loss of epithelium on the mucosal surface; and there was inflammatory cell infiltration (Figure 5A,B). This may be caused by the intestinal infection with *E. coli*. To evaluate the therapeutic efficacy of enrofloxacin-loaded COS–SA composite core-shell nanogels on intestinal bacterial infection caused by *E. coli*, the curing rates were calculated. The treatments were categorized into the NC group, IC group, Native 1 group, and Native 2 group. The curing of the infected mice was determined by histological analyses (Figure 5C–F). When there were no pathological changes in the intestinal tissue, it was observed that the infected mice were completely treated. The curing rates of the Native 1 group and Native 2 group were 73.3% and 80.0%, respectively. These suggested that the same dose of enrofloxacin-loaded COS–SA composite nanogels could achieve a better treatment effect compared to that of commercial enrofloxacin solutions.

### 3.6. Biosafety Studies

**Figure 6:**
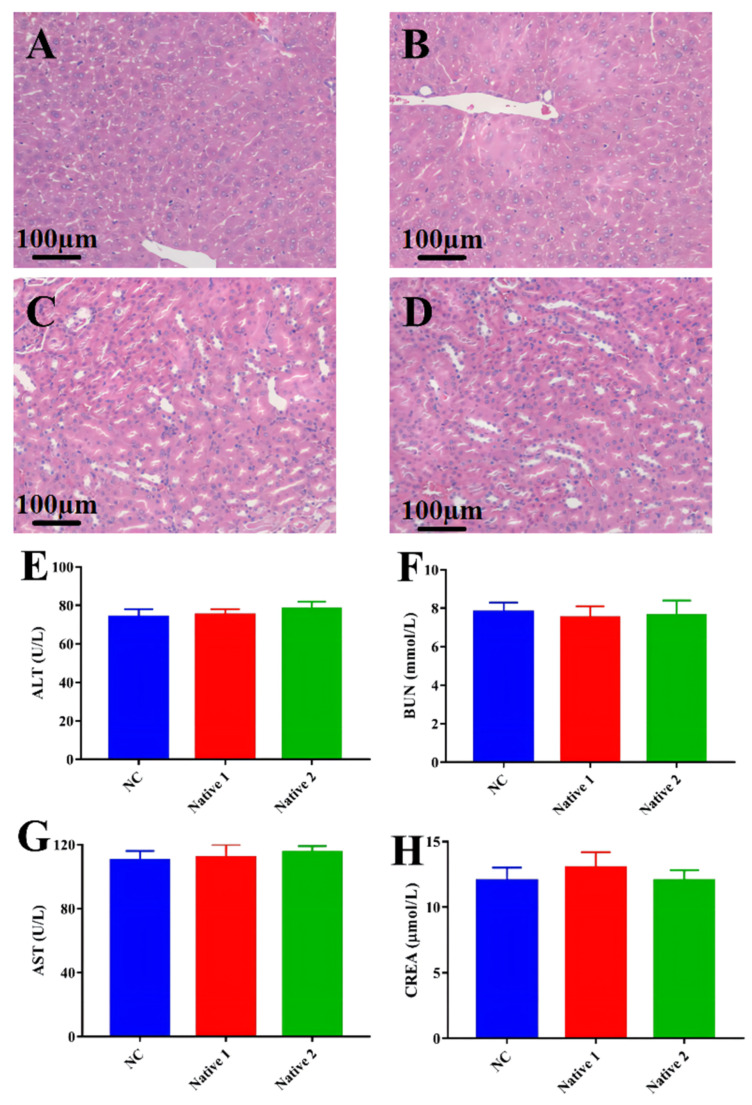
Biosafety studies. The histological evaluation of mice treated with enrofloxacin composite nanogels group (Magnification 200×). (**A**): liver of infected mice treated with enrofloxacin composite nanogels; (**B**): liver of non-infected mice; (**C**): kidney of infected mice treated with enrofloxacin composite nanogels; (**D**): kidney of non-infected mice. (**E**–**H**) Rsesults of blood biochemical parameters (ALT, BUN, AST and CREA) in NC, group Native 1, and group Native 2 (Mean ± SD, *n* = 3).

As the intelligent-responsive enrofloxacin composite nanogels are applied to the intestinal bacterial infection caused by *E. coli*, their biocompatibility is regarded as the most essential aspect to be concerned. Thus, the biosafety of the enrofloxacin composite nanogels was evaluated via H&E staining and an in vivo blood biochemical analysis. Compared with the NC group, there were no significant pathological changes in mice gavaged with enrofloxacin composite nanogels (Figure 6A,B). The blood biochemical parameters were within normal limits without showing any significant differences (Figure 6C–F), ensuring that the enrofloxacin core-shell nanogels had no significant systemic toxic effects on the hepatic and renal functions. These findings suggested that the enrofloxacin composite nanogels could be a great promising biocompatible intelligent-responsive compound for curing intestinal bacterial infections caused by *E. coli*.

## 4. Conclusions

The COS–SA composite core-shell nanogels loaded with enrofloxacin were used to study the therapeutic effects of enrofloxacin against intestinal bacterial infections caused by *E. coli*. Through characterization and an FTIR study, the enrofloxacin composite nanogels were successfully formulated. The prepared enrofloxacin composite nanogels ensured that the enrofloxacin had stability in the stomach and was slowly released in the intestinal tract. The antibacterial activity study demonstrated that enrofloxacin core-shell nanogels displayed ideal and effectively dynamic antibacterial activity against *E. coli*. Further, the same dose of enrofloxacin nanogels could reach a better treatment effect than that of the commercial enrofloxacin solution. Furthermore, the biosafety studies showed great promise for the enrofloxacin composite nanogels as a biocompatible, intelligent-responsive composition for intestinal bacterial infections caused by *E. coli*. Subsequently, we suggest that the provided enrofloxacin nanogels could be an intelligent-responsive preparation with on-demand release and an excellent palatability to effectively overcome the treatment constraints of intestinal bacterial infections presented by *E. coli*.

## Figures and Tables

**Table 1:** Optimization of chitosan oligosaccharide–sodium alginate composite core-shell nanogels loaded with enrofloxacin (Mean ± SD, *n* = 3).

Chitosan Oligosaccharide (mg/mL)	Sodium Alginate (mg/mL)	CaCl_2_ (mg/mL)	Enrofloxacin (mg/mL)	Loading Capacity (%)	Encapsulation Efficiency (%)
12	2	0.1	5	15.3 ± 0.8	63.7 ± 0.7
4	2	0.2	15	18.5 ± 0.3	65.2 ± 0.4
8	2	0.4	10	25.7 ± 0.9	68.3 ± 0.2
8	8	0.2	5	26.6 ± 0.5	72.4 ± 0.8
12	4	0.2	10	21.5 ± 1.3	71.3 ± 1.3
8	4	01	15	20.1 ± 0.5	65.8 ± 0.5
4	2	0.1	10	21.4 ± 0.4	61.4 ± 0.8
12	8	0.4	15	20.1 ± 1.7	71.4 ± 0.9
4	8	0.1	10	23.9 ± 1.5	63.2 ± 1.1

**Table 2:** The mean particle diameter, polydispersity index, and zeta potential of the enrofloxacin-loaded chitosan oligosaccharide–sodium alginate composite core-shell nanogels (*n* = 3) at distinct pH environments (Mean ± SD).

pH Value	Mean Particles Diameter (nm)	Polydispersity Index	Zeta Potential (mV)
1.2	168.6 ± 4.2	0.24 ± 0.05	38.7 ± 0.75
7.4	134.4 ± 3.2	0.65 ± 0.03	−36.5 ± 0.43

## Data Availability

Data will be made available upon reasonable request.
